# Perceiving a need for dietary change in adults living with and beyond cancer: A cross‐sectional study

**DOI:** 10.1002/cam4.7073

**Published:** 2024-03-08

**Authors:** Susan Smith, Abi Fisher, Phillippa J. Lally, Helen A. Croker, Anna Roberts, Rana E. Conway, Rebecca J. Beeken

**Affiliations:** ^1^ Department of Behavioural Science and Health University College London London UK; ^2^ Department of Psychology University of Surrey Surrey UK; ^3^ Leeds Institute of Health Sciences University of Leeds Leeds UK

**Keywords:** cancer survivors, diet, diet quality, energy intake, healthy diet, nutrition surveys

## Abstract

**Background:**

Many people living with and beyond cancer (LWBC) do not meet dietary recommendations. To implement a healthier diet, people LWBC must perceive a need to improve their diet.

**Methods:**

Participants included people diagnosed with breast, prostate or colorectal cancer in the UK. Two binary logistic regression models were conducted with perceived need for dietary change as the outcome (need to improve vs. no need). Predictor variables included demographic and clinical characteristics, receipt of dietary advice, and either body mass index (BMI) or adherence to seven relevant World Cancer Research Fund (WCRF) dietary recommendations.

**Results:**

The sample included 5835 responses. Only 31% perceived a need to improve their diet. Being younger (odds ratio [OR] 0.95, 95% confidence interval [CI] = 94–0.95), female (OR = 1.33, 95% CI = 1.15–1.53), not of white ethnicity (OR = 1.8, 95% CI = 1.48–2.27), not married/cohabiting (OR = 1.32, 95% CI = 1.16–1.52) and having received dietary advice (OR = 1.36, 95% CI = 1.43–1.86) was associated with an increased odds of perceiving a need to improve diet. This association was also seen for participants with two or more comorbidities (OR = 1.31, 95% CI = 1.09–1.57), those not meeting the recommendations for fruit and vegetables (OR = 0.47, 95% CI = 0.41–0.55), fat (OR = 0.67, 95% CI = 0.58–0.77), and sugar (OR = 0.86, 95% CI = 0.75–0.98) in the dietary components model and those who had a higher BMI (OR = 1.53, 95% CI = 1.32–1.77) in the BMI model.

**Conclusions:**

Most of this sample of people LWBC did not perceive a need to improve their diet. More research is needed to understand the reasons for this and to target these reasons in dietary interventions.

## Graphical abstract

Despite published dietary recommendations, we found that many people living with and beyond cancer do not follow a healthy diet and only 31% of our sample (*n* = 5835) perceived a need to improve their diet. Being younger, female, not of white ethnicity, not married/cohabiting, having more comorbidities, having a higher body mass index, and not meeting various dietary recommendations was associated with perceiving a need to improve diet.

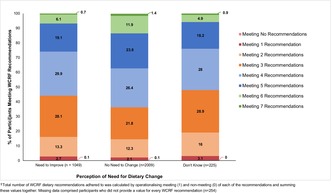

## 1 INTRODUCTION

Advancements in the detection and treatment of cancer have led to improvements in cancer survival rates worldwide.^1^ Current cancer research and care look beyond treatment and survival as key outcomes following a diagnosis and aim to support people living with and beyond cancer (LWBC) to maintain a good quality of life and make healthier lifestyle choices to improve their long‐term health.^2^, ^3^, ^4^ In this context, people LWBC encompasses all people, who have been diagnosed with cancer, without distinction between those who are still receiving or have completed treatment.

To support this population, the World Cancer Research Fund (WCRF) published lifestyle recommendations for people LWBC, including several diet‐related recommendations.^5^ The recommended diet is characterised by increased consumption of fruit, vegetables and fibre, alongside reduced consumption of red meat, processed meat, calorie‐dense, nutrient‐poor foods and alcohol.^6^, ^7^, ^8^, ^9^ This includes consuming a diet with at least five portions of fruit and vegetables per day and at least 30 g of fibre per day, while limiting consumption of meat to less than 500 g of red meat per week and avoiding processed meat.^5^ Additionally, the WCRF recommends limiting the consumption of foods high in fat and sugar, sugar sweetened drinks and alcohol.^5^ These recommendations are grounded in limited, but consistent evidence demonstrating that a healthy diet is associated with improvements in both cancer‐related and non‐cancer‐related outcomes.^10^, ^11^, ^12^, ^13^ Recent meta‐analyses have demonstrated a reduced risk for all‐cause mortality and cancer mortality, as well as cancer recurrence in people LWBC who demonstrate better diet quality.^13^, ^14^, ^15^, ^16^ Beyond survival alone, cross‐sectional studies suggest that improved adherence to dietary recommendations in people LWBC is associated with improved psychosocial outcomes, such as health‐related quality of life.^17^, ^18^, ^19^ While Aldossari et al. reported in their systematic review of 14 observational studies that people make positive changes to their diet after a cancer diagnosis, these changes tend to be minimal and not clinically meaningful,^20^ few studies have compared pre‐diagnosis and post‐diagnosis diet quality to explore the impact of changing diet on survival and other health outcomes.^21^, ^22^ In their study of 2295 postmenopausal women diagnosed with breast cancer, Sun et al. reported that a decrease in diet quality after diagnosis was associated with a higher risk of death from breast cancer, but improving diet quality did not demonstrate a lower mortality risk, although this could be due to improvements being too modest to impact mortality risk.^22^

Several studies have operationalised adherence to the WCRF recommendations and generally report low adherence rates.^10^, ^23^, ^24^, ^25^, ^26^, ^27^, ^28^ While compliance to fruit and vegetable recommendations is the most commonly reported indicator of dietary quality,^23^, ^24^ Winkels and colleagues also reported poor adherence to the recommendations for meat, fibre and energy‐dense nutrient‐poor foods in 1196 people diagnosed with colorectal cancer.^27^ Similar findings were observed in a small sample of women diagnosed with breast cancer, although better adherence to the red meat recommendations was found.^28^ However, low adherence rates may be explained by limited knowledge about what constitutes a healthy diet. Despite a documented desire for dietary support and advice in this population,^29^, ^30^ a recent scoping review by Johnston and colleagues reported that the dietary needs and preferences of people LWBC do not align with their access to guidance and information.^31^ People LWBC may not recognise that their diets are sub‐optimal,^30^, ^32^ with data from qualitative research suggesting that a belief that one's diet is already healthy enough is a barrier to instigating healthful dietary changes in people LWBC.^30^ There is a documented ‘optimism bias’ in dietary perceptions, where people overestimate the healthfulness of their diet.^33^, ^34^ This misperception is exhibited in the general population,^35^, ^36^ and in clinical populations, including people LWBC^37^, ^38^ and may mean that individuals do not believe in, nor identify a need to, improve their diet.^34^, ^35^ This has been observed in a nationally representative sample of people LWBC where Xue et al. reported low conformance between perceived and actual dietary quality, with 56% of participants being categorised as incorrectly optimistic about their diets.^38^

Many sociodemographic, clinical and dietary factors might contribute to these misperceptions of dietary quality.^35^ In Xue et al.'s study, each 10‐year increase in age was associated with greater odds of being incorrectly optimistic about dietary quality,^38^ while different age groups had a differential effect on being incorrectly optimistic in Variyam et al.'s study of the general population.^36^ While there are only few studies examining predictors of misperceptions of dietary quality,^36^, ^38^ previous research is inconclusive regarding associations between dietary quality perception (whether correct or not) and demographic factors such as age^38^, ^39^, ^40^, ^41^ and sex.^37^, ^38^, ^40^ While, Batis et al. and Gago et al. observed similarities in the mean ages of participants across differing perceptions of dietary quality,^40^, ^41^ Sullivan et al. reported that adults, who perceive their diet to be of higher quality tend to be older than those who report lower self‐ratings of dietary quality.^39^ However, these discrepancies may be attributed to differences in how perceived dietary quality is measured across studies, either dichotomously^40^ or using rating scales.^39^, ^41^ Further research suggests that individuals with higher education levels tend to self‐report better dietary quality,^39^ but the accuracy of this is compromised by Xue et al.'s observation that this group is also more likely to demonstrate an optimism bias.^38^ There is more consistent evidence that people with overweight and obesity tend to perceive their dietary quality to be poorer than those in healthier weight categories.^39^, ^40^ Similarly, when asked if they consider their diet to be healthy, people who rate their dietary quality as healthier (without investigation of the accuracy of this perception) tend to demonstrate significantly higher intake of fruits and vegetables, fish, and fibre^37^, ^40^ and lower intake of sugary and salty foods.^40^ However, there has been little research on whether similar factors are associated with a perceived need to change diet.

The importance of investigating perceptions surrounding a need for dietary change is underscored in Michie et al.'s capability, opportunity and motivation model of behaviour (COM‐B).^42^ As part of their psychological capability, the individual must first be able to evaluate their current behaviour against the potential benefits of modifying this behaviour. Recognising that there are areas for improvement in their diet quality and therefore, perceiving a need to improve diet is then a contributing factor in determining whether a change in dietary behaviour will take place and can influence the behaviour directly, or indirectly via motivation. The current study therefore aimed to identify the proportion of adults LWBC perceiving a need to improve diet and to investigate factors influencing this perception, including demographic and clinical characteristics, body mass index (BMI) and intake of specific dietary components.

## 2 METHODS

### 2.1 Design

Secondary analysis was conducted on the cross‐sectional data collected during the pre‐trial stage of the Advancing Survival after Cancer Outcomes Trial (ASCOT).^43^ ASCOT is a randomised controlled trial investigating a brief lifestyle advice intervention in people diagnosed with breast, prostate or colorectal cancer that began recruitment in 2015.^43^ The intervention targeted nine health behaviours including physical activity, diet, alcohol and smoking, where a change in a calculated composite health behaviour risk index was the primary outcome of the trial. The ‘Health and Lifestyle After Cancer Survey’ was used to identify initial interest in the ASCOT trial and comprised questions about demographics, clinical characteristics and health behaviours in people LWBC.

### 2.2 Procedure

Eligible participants for the ‘Health and Lifestyle After Cancer’ questionnaire were identified via 10 participating NHS hospital sites across London and Essex. Patients who had received a diagnosis of breast, colorectal or prostate cancer between 2012 and 2015 were mailed the survey pack. These dates were chosen as the survey was used to identify interest in the ASCOT trial, where participants were only patients who had completed primary curative treatment.^43^ Packs were sent out between February 2015 and November 2017 and returned questionnaires were accepted until the 4th of January 2018. Ethical approval was obtained through the National Research Ethics Service Committee South Central—Oxford B (reference number 14/SC/1369).

### 2.3 Participants

Participants in this study were over 18 years old, had received a primary diagnosis of breast, prostate or colorectal cancer at the participating hospital sites and were able to provide consent for themselves and were without cognitive impairment. Although the sample was primarily comprised of patients diagnosed between 2012 and 2015, the final sample in the analysis included patients diagnosed outside of these dates (range: 1994–2017). The recorded date of diagnosis was that of their most recent diagnosis of breast, prostate or colorectal cancer, as some participants had received more than one diagnosis. Exclusion criteria (patient deceased or deemed inappropriate to contact) were intentionally limited to maximise the reach of the study and to minimise the burden of survey administration at sites.

### 2.4 Measures

#### 2.4.1 Demographic characteristics

Participants were asked to self‐report age (in years), sex (male, female), marital status (married or living with partner, separated, divorced, widowed, single), and education level (ranging from ‘no formal qualifications’ to ‘Masters/PhD/Postgraduate Certificate in Education (PGCE) or equivalent’). Marital status was collapsed into two categories due to small numbers in the non‐married/cohabiting groups (married/cohabiting, separated/divorced/widowed/single). Education level was collapsed into four categories (no formal qualifications, General Certificate of Secondary Education (GCSE)/vocational, A‐level, degree or higher). A‐levels are equivalent to school leaving qualifications such as the High School Diploma. Ethnicity information was collected by 15 possible responses to the question ‘Which of these best describes your ethnic group?’ and collapsed into two categories (white, any other ethnicity) as numbers in the other ethnicity categories were low (9.5%).

#### 2.4.2 Clinical characteristics

The questionnaire asked participants ‘Which of these types of cancer have you been diagnosed with?’ (breast, prostate, bowel [colorectal], other) and whether it had spread to any other parts of their body. Cancer type was recorded as the most recent out of the three cancer types that was reported by participants. Time since diagnosis (in months) was based on participants' date of their most recent diagnosis of breast, prostate, or colorectal cancer and the date the questionnaire was received back at the university. Treatment type was assessed by ‘What treatment(s) have you had for this cancer? Please tick all that apply’ (surgery, radiotherapy, chemotherapy, hormone therapy, active surveillance, none, not sure), meaning some participants selected multiple treatment types. As many participants specified biological therapy under the ‘other’ category, we created an additional category specifically for this treatment type. BMI was calculated from self‐reported height and weight. Participants were classified as underweight (BMI < 18.5), healthy (BMI ≥ 18.5 and < 25), overweight (BMI ≥ 25 and < 30) or obese (BMI ≥ 30).^44^ The ‘underweight’ category was combined with the ‘healthy’ category due to low numbers (1.36%). To assess the number of comorbid conditions, participants were asked to tick all that applied in response to ‘Have you ever had any of the following health problems?’: osteoporosis, diabetes, asthma, emotional or psychiatric illness, stroke, Parkinson's disease, Alzheimer's disease or dementia, lung disease, arthritis, angina, heart attack, heart murmur, irregular heart rhythm, any other heart trouble, another cancer, or other health problems not listed. Number of comorbid conditions was collapsed into four categories (0, 1, 2, ≥3).

#### 2.4.3 Dietary advice

Participants were presented with the question ‘In the time since you were first diagnosed with cancer, did a health professional ever recommend any of the following?’ (yes, no). The options included ‘Eating more fruit and vegetables’, ‘Avoiding foods or drinks high in fat, sugar or salt’, ‘Eating less red or processed meat’ and ‘Reducing the amount of alcohol you drink’. If participants answered yes to any of these, they were classified as having received dietary advice.

#### 2.4.4 Dietary components

Seven relevant dietary components were assessed using an adaptation of the Dietary Instrument for Nutrition Education Food Frequency Questionnaire (DINE FFQ) asking about participants' current diet.^45^ The DINE FFQ has been validated in the general population^45^ and was chosen after a review of validated food frequency questionnaires and a review of how diet was assessed in previous studies of people LWBC.^43^ Based on a review of the National Diet and Nutrition Survey and the Low Income Diet and Nutrition Survey, some food items were updated to ensure that the UK diet was reflected in the items. The adapted DINE FFQ therefore included more ethnically diverse foods that are presently available, as well as enabling estimations of the relevant WCRF diet components.^43^ The items used to estimate intake of each component have previously been described for this dataset.^46^ The seven dietary components in this study were selected based on the WCRF/AICR recommendations and in line with the national United Kingdom (UK) recommendations^46^, ^47^ and included the recommendations for the intake of fibre (≥30 g per day),^5^, ^48^ fruit and vegetables (≥5 portions [400 g] per day),^5^ red meat (<500 g per week),^5^ processed meat (none),^5^ fat (<33% of calories from fat per day),^5^, ^49^ sugar (<5% of calories from free sugars per day),^5^, ^48^ and alcohol (≤14 units per week).^5^, ^50^ Adherence to each of these recommendations was operationalised using a scoring system implemented and described previously for this trial.^46^

#### 2.4.5 Perception of the need for dietary change

Participants' perception of the need for dietary change was assessed using the question ‘Which of the following best describes you at the present time?’. Response options included ‘I think I should have a healthier diet’; ‘I don't think I need to change my diet’; ‘Don't know’. This item was custom‐made for the study.

### 2.5 Missing data

Multiple imputation in SPSS was used to handle missing data and to reduce bias introduced by incomplete cases. The imputation model included all variables in the analysis. Missing data analysis found that 65.3% of cases had missing data, with 4.8% of 332,595 values missing for the included variables. Little's *t*‐test determined that these were not missing completely at random. Imputation was performed on the dietary perception variable (1.1% missing), demographic and clinical variables: age (0.6% missing), sex (0.3% missing), ethnicity (0.5% missing), marital status (0.3% missing), and highest education level (9.4% missing), height (2.3% missing) and weight (4.4% missing) variables used to calculate BMI, the cancer spread variable (13.4% missing), and time since diagnosis (0.6% missing). Number of comorbidities contained no missing data. Each dietary advice variable was included: eating more fruit and vegetables (9.7% missing), avoiding foods high in sugar and fat (10.7% missing), eating less red and processed meat (11.9% missing), and reducing alcohol (14.9% missing). All scale item variables assessing dietary intake were included in the imputation (41 variables; 4.6% missing values) before recalculation of total intake scores to determine meeting/non‐meeting WCRF recommendations. The standard five imputations were conducted with 10 iterations per imputation.^51^ After running the imputation model twice and running the analyses with both datasets, the results were similar and considered to converge and therefore five imputations were considered adequate. Results from the first imputation model are reported.

### 2.6 Statistical analysis

All statistical analyses were performed using SPSS version 26. Two separate binary logistic regression models were conducted on the imputed dataset to determine factors that influence perceptions of need for dietary change (need to improve vs. no need to change). As BMI may mediate any association between dietary intake and perceptions of the need for dietary change and Tennant et al.^52^ advises against controlling for potential mediators, separate models investigated the role of dietary components and perceiving a need to improve diet, and BMI and perceiving a need to improve diet. A mediation analysis could not be conducted as temporal ordering could not be determined in this cross‐sectional data.^53^ Missing values were imputed at this level. Respondents who selected ‘don't know’ were coded as missing before conducting the regression analyses since it was not appropriate to impute where true values were given but it was not possible to include this group in the regression model due to small numbers (8%). Demographic and clinical characteristics including age, gender, ethnicity, marital status, education, cancer spread, time since diagnosis, number of comorbidities and receipt of dietary advice were entered into the model simultaneously with either the seven relevant dietary components (fibre, fruit and vegetables, red meat, processed meat, fat, sugar, and alcohol) or BMI. Cancer type was not included in the regression models due to collinearity between cancer type and sex. Supplementary stratified analyses by cancer type were conducted in addition to the main analyses. Both regression analyses were repeated with the non‐imputed data to explore, whether findings were similar in the original data.

## 3 RESULTS

### 3.1 Sample characteristics

Of 13,500 surveys mailed to potential participants, 5835 were returned (43% response rate). Most participants perceived no need to change their diet (3511; 60%), while 1793 (31%) perceived a need to improve their diet, and 468 (8%) participants reported not knowing. Only 63 (1%) participants had missing data for this variable.

Table 1 presents the characteristics of the people included in this study. The mean age of participants was 67.43 (SD = 11.8, range 26–97). Participants were mainly of white ethnicity (90%, *n* = 5249) with slightly more females (56%, *n =* 3266). Cancer diagnoses included breast cancer (48%, *n =* 2786), prostate cancer (32%, *n =* 1839) and colorectal cancer (21%, *n =* 1210). Participants were categorised into healthy/underweight (35%, *n =* 2043), overweight (39%, *n* = 2247), and obese (21%, *n =* 1209) groups according to BMI classifications. Adherence to the WCRF recommendations was mixed, with the highest rates of adherence observed for the red meat (86%, *n =* 5035) and alcohol (83%, *n =* 4848) recommendations.

**TABLE 1:** Sample characteristics according to perception of diet in the original data.

	Total (*n* = 5835)	Perception of diet, *n* (valid %)^a^		
		Need to improve *(n* = 1793, 30.2)	No change needed (*n* = 3511, 60.2)	Don't know (*n =* 468, 8)
Age, mean (SD)	67.4 (11.8)	62.9 (11.4)	69.5 (11.3)	69.1 (12.3)
Missing, *n* (%)	36 (0.6)			
Sex, *n* (%)				
Male	2553 (43.8)	632 (35.3)	1684 (48.1)	209 (44.8)
Female	3266 (56.0)	1157 (64.7)	1818 (51.9)	258 (55.2)
Missing	16 (0.3)			
Ethnicity, *n* (%)				
White	5249 (90)	1525 (85.5)	3259 (93.4)	407 (87.5)
Any other ethnicity	554 (9.5)	259 (14.5)	232 (6.6)	58 (12.5)
Missing	32 (0.5)			
Highest level of education, *n* (%)				
None	1709 (29.3)	420 (25.2)	1076 (34.2)	189 (45.0)
GCSE/vocational	1613 (27.6)	553 (33.2)	937 (29.8)	109 (26.0)
A‐level	584 (10)	206 (12.4)	329 (10.5)	44 (10.5)
Degree	1379 (23.6)	488 (29.3)	806 (25.6)	78 (18.6)
Missing	550 (9.4)			
Marital status, *n* (%)				
Married/cohabiting	4037 (69.2)	1194 (66.8)	2529 (72.2)	279 (59.7)
Separated/divorced/widowed/single	1781 (30.5)	594 (33.2)	974 (27.8)	188 (40.3)
Missing	17 (0.3)			
BMI, *n* (%)				
Underweight/healthy	2043 (35)	490 (28.9)	1394 (41.8)	136 (32.9)
Overweight	2247 (38.5)	680 (40.1)	1396 (41.8)	153 (37.0)
Obese	1209 (20.7)	527 (31.1)	546 (16.4)	125 (30.2)
Missing	336 (5.8)			
Cancer type, *n* (%)				
Breast	2786 (47.7)	991 (55.3)	1513 (43.1)	203 (43.4)
Prostate	1839 (31.5)	474 (26.4)	1170 (33.3)	140 (29.9)
Colorectal	1210 (20.7)	288 (16.1)	738 (21.0)	113 (24.3)
Treatment type, *n* (%)				
Surgery	4065 (69.7)	1351 (76.2)	2361 (68.1)	314 (68.6)
Radiotherapy	3348 (57.4)	1109 (62.6)	1954 (56.3)	263 (57.0)
Chemotherapy	1824 (31.3)	689 (39.0)	966 (28.0)	148 (32.4)
Hormone therapy	1895 (32.5)	621 (35.1)	1128 (32.6)	132 (28.9)
Active surveillance	1017 (17.4)	292 (16.5)	644 (18.6)	68 (14.8)
Biological therapies	123 (2.1)	49 (2.8)	58 (1.7)	13 (2.9)
Other treatment	46 (0.8)	13 (0.7)	28 (0.8)	4 (0.9)
No treatment	113 (1.9)	28 (1.6)	69 (2.0)	13 (2.9)
Missing	61 (1.0)			
Months since diagnosis, mean (SD)	36.1 (13.8)	35.8 (12.7)	36.1 (14.5)	37.1 (13.4)
Cancer spread, *n* (%)				
Yes	558 (9.6)	183 (11.6)	319 (10.5)	52 (13.5)
No	4498 (77.1)	1389 (88.4)	2730 (89.5)	332 (86.5)
Missing/don't know	779 (13.4)			
Number of comorbid conditions, *n* (%)				
0	1849 (31.7)	613 (34.2)	1096 (31.2)	117 (25.0)
1	1991 (34.1)	579 (32.3)	1252 (35.7)	143 (30.6)
2	1120 (19.2)	336 (18.7)	658 (18.7)	112 (23.9)
3 or more	875 (15.0)	265 (14.8)	505 (14.4)	96 (20.5)
Receipt of dietary advice, *n* (%)				
No advice received	3352 (57.4)	923 (62.3)	2152 (72.8)	246 (68.5)
Advice received	1486 (35.1)	558 (37.7)	804 (27.2)	113 (31.5)
Missing	997 (7.5)			
WCRF/AICR fibre, *n* (%)				
Not meeting	3900 (85.4)	1268 (87.3)	2342 (84.2)	290 (87.3)
Meeting	666 (14.6)	184 (12.7)	440 (15.8)	42 (12.7)
Missing	1269 (21.7)			
WCRF/AICR fruit and vegetables, *n* (%)				
Not meeting	3914 (67.1)	1365 (77.5)	2242 (65.5)	373 (83.6)
Meeting	667 (11.4)	391 (22.5)	1181 (34.5)	73 (16.4)
Missing	1254 (21.5)			
WCRF/AICR red meat, *n* (%)				
Not meeting	139 (2.4)	35 (2.1)	80 (2.6)	22 (5.6)
Meeting	5035 (86.3)	1595 (97.9)	3035 (97.4)	371 (94.4)
Missing	661 (11.3)			
WCRF/AICR processed meat, *n* (%)				
Not meeting	2861 (49.0)	856 (50.4)	1750 (52.5)	232 (54.6)
Meeting	2640 (45.2)	844 (49.6)	1583 (47.5)	193 (45.4)
Missing	334 (5.7)			
WCRF/AICR sugar, *n* (%)				
Not meeting	2663 (45.6)	863 (52.1)	1548 (47.8)	224 (54.8)
Meeting	2694 (46.2)	794 (47.9)	1693 (52.2)	185 (45.2)
Missing	478 (8.2)			
WCRF/AICR fat, *n* (%)				
Not meeting	1769 (30.3)	594 (46.5)	1038 (41.9)	130 (45.3)
Meeting	2300 (39.4)	684 (53.5)	1442 (58.1)	157 (54.7)
Missing	1766 (30.3)			
WCRF/AICR alcohol, *n* (%)				
Not meeting	714 (12.2)	207 (12.0)	457 (13.6)	46 (10.5)
Meeting	4848 (83.1)	1516 (88.0)	2904 (86.4)	392 (89.5)
Missing	273 (4.7)			

Abbreviations: A‐level, General Certificate of Education Advanced Level; BMI, body mass index; GCSE, General Certificate of Secondary Education; WCRF/AICR, World Cancer Research Fund and American Institute for Cancer Research recommendations.

^a^ Diet perception values missing for 1.1% of total sample (*n* = 63).^46^

**FIGURE 1:**
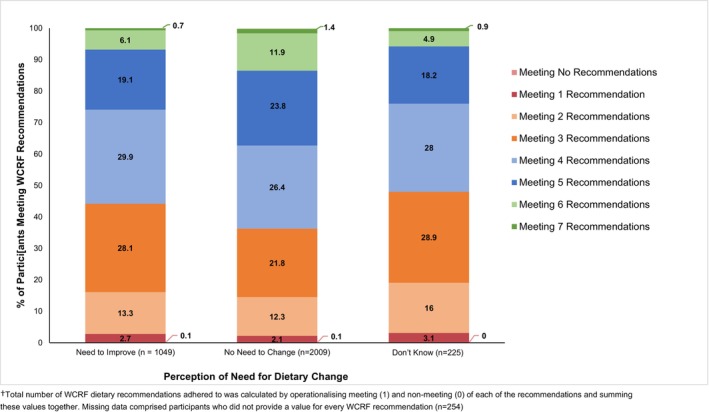
Adherence to World Cancer Research Fund (WCRF) dietary recommendations by perceptions of need for dietary change^a^. ^a^Total number of WCRF dietary recommendations adhered to was calculated by operationalising meeting (1) and non‐meeting (0) of each of the recommendations and summing these values together. Missing data comprised participants, who did not provide a value for every WCRF recommendation (*n* = 254).

Figure 1 presents adherence to the WCRF recommendations according to perceptions of need for dietary change in the original data. Overall, 1.2% of the sample met all seven WCRF recommendations. In those perceiving no need to change their diet, 1.4% met all the recommendations.

### 3.2 Characteristics associated with perceiving a need for dietary change

Tables 2 and 3 present the binary logistic regression analyses examining associations between demographic characteristics, clinical characteristics, receipt of dietary advice and BMI (Table 2) or dietary components (Table 3) in perceptions of need for dietary change, where perceiving a need to improve diet was the reference group. Associations between perceptions of need for dietary change and demographic and clinical characteristics, and receipt of dietary advice were similar in both models. Odds ratios and confidence intervals are reported from the model investigating dietary components. Stratified analyses by cancer type demonstrated broadly similar findings across the three cancer types and are presented in the supporting information.

**TABLE 2:** Pooled (five imputations) multivariate logistic regression analysis for the association between body mass index (BMI) and perceiving a need to improve diet (*n* = 5356)^a^.

	OR	95% CI	*p*
Age	0.95	0.95–0.96	<0.001*
Sex			
Male	1		
Female	1.21	1.06–1.39	0.006*
Education level			
None	1		
GCSE/vocational	1.01	0.84–1.21	0.959
A‐level	0.99	0.79–1.23	0.953
Degree and above	0.99	0.83–1.19	0.936
Ethnicity			
White	1		
Any other ethnicity	1.78	1.44–2.18	<0.001*
Marital status			
Married/cohabiting	1		
Separated/divorced/widowed/single	1.37	1.20–1.57	<0.001*
Cancer spread			
Yes	1		
No	1.11	0.90–1.36	0.335
Months since cancer diagnosis	1.00^b^	0.99–1.00	0.625
Number of comorbid conditions			
None	1		
1	0.97	0.83–1.13	0.690
2	1.16	0.97–1.40	0.104
3 or more	1.21	0.99–1.48	0.068
Receipt of dietary advice			
No advice received	1		
Advice received	1.54	1.35–1.76	<0.001*
BMI			
Healthy/underweight	1		
Overweight	1.53	1.32–1.77	<0.001*
Obese	2.73	2.31–3.24	<0.001*

Abbreviations: 95% CI, 95% confidence interval; A‐level, General Certificate of Education Advanced Level; BMI, body mass index; GCSE, General Certificate of Secondary Education; OR, odds ratio.

* Statistical significance at *p* < 0.05.

^a^ ‘Don't know’ cases excluded from analysis.

^b^ 0.999 rounded up.

**TABLE 3:** Pooled (five imputations) multivariate logistic regression analysis for the association between dietary components and perceiving a need to improve diet (*n* = 5356)^a^.

	OR	95% CI	*p*
Age	0.95	0.94–0.95	<0.001*
Sex			
Male	1		
Female	1.33	1.15–1.53	<0.001*
Education level			
None	1		
GCSE/vocational	1.02	0.85–1.22	0.861
A level	1.02	0.81–1.28	0.885
Degree and above	1.04	0.86–1.25	0.707
Ethnicity			
White	1		
Any other ethnicity	1.83	1.48–2.27	<0.001*
Marital status			
Married/cohabiting	1		
Separated/divorced/widowed/single	1.32	1.16–1.52	<0.001*
Cancer spread			
Yes	1		
No	1.14	0.93–1.40	0.222
Months since diagnosis	1^b^	0.99–1.00	0.272
Number of comorbid conditions			
None	1		
1	1.03	0.89–1.20	0.673
2	1.31	1.09–1.57	0.004*
3 or more	1.43	1.17–1.75	<0.001*
Dietary advice received			
No advice received	1		
Advice received	1.63	1.43–1.86	<0.001*
WCRF/AICR fibre			
Not meeting	1		
Meeting	0.91	0.75–1.10	0.315
WCRF/AICR fruit and vegetables			
Not meeting	1		
Meeting	0.47	0.41–0.55	<0.001*
WCRF/AICR red meat			
Not meeting	1		
Meeting	1.32	0.90–1.94	0.138
WCRF/AICR processed meat			
Not meeting	1		
Meeting	0.94	0.82–1.08	0.375
WCRF/AICR fat			
Not meeting	1		
Meeting	0.67	0.58–0.77	<0.001*
WCRF/AICR sugar			
Not meeting	1		
Meeting	0.86	0.75–0.98	0.019*
WCRF/AICR alcohol			
Not meeting	1		
Meeting	1.12	0.92–1.37	0.263

Abbreviations: 95% CI, 95% confidence interval; A‐level, General Secondary School Advanced Level; GCSE, General Certificate of Secondary Education; OR, odds ratio; WCRF/AICR, World Cancer Research Fund and American Institute for Cancer Research recommendations.

* Statistical significance at *p* < 0.05.

^a^ ‘Don't know’ cases excluded from analysis.

^b^ 0.997 rounded up.

#### 3.2.1 Demographic characteristics

Age was associated with a perceived need to improve their diet, with older participants less likely to perceive this need. Specifically, every year increase in age was associated with 5% lower odds of perceiving a need to improve diet (OR = 0.95, 95% CI 0.94–0.95). Female participants had greater odds of perceiving a need to improve their diet than males (OR = 1.33, 95% CI = 1.15–1.53). Compared to participants of white ethnicity, participants of any other ethnicity had greater odds of perceiving a need to improve diet (OR = 1.83, 95% CI = 1.48–2.27). Participants who were not married/cohabiting had greater odds of perceiving a need to improve their diet (OR = 1.32, 95% CI = 1.16–1.32) Education level was not associated with perceptions of need for dietary change.

#### 3.2.2 Clinical characteristics

The number of comorbid conditions was significantly associated with perceptions of need for dietary change. Compared to participants with no comorbidities, participants with two (OR = 1.31, 95% CI = 1.09–1.57) or three or more comorbidities (OR = 1.43, 95% CI = 1.16–1.75) demonstrated greater odds of perceiving a need to improve their diet. Cancer spread and time since diagnosis were not associated with perceiving a need to change diet.

#### 3.2.3 Receipt of dietary advice

Participants, who had received dietary advice were more likely to perceive a need to improve their diet (OR = 1.63, 95% CI = 1.43–1.86).

#### 3.2.4 Body mass index

Participants classified as overweight had 50% greater odds of perceiving a need to improve their diet compared to participants in the healthy/underweight category (OR = 1.53, 95% CI = 1.32–1.77). The odds of perceiving a need to improve diet were more than doubled in participants with obesity (OR = 2.73, 95% CI = 2.31–3.24).

#### 3.2.5 Dietary components

Participants, who met the WCRF/AICR recommendations for fruit and vegetables (OR = 0.47, 95% CI = 0.41–0.55), fat (0.67, 95% CI = 0.58–0.77) and sugar (OR = 0.86, 95% CI = 0.75–0.98) demonstrated lower odds of perceiving a need to improve their diet, compared to those who did not meet recommendations. Meeting the recommendations for fibre, red meat, processed meat, and alcohol were not associated with perceiving a need to change diet.

### 3.3 Analysis with original data

Descriptive statistics for participants, who provided responses on all key variables (completers) compared to those who had missing data (non‐completers), alongside results from the logistic regression analyses with the complete case data, are presented in the supporting information, showing similar patterns to the imputed data. A comparison of people, who were included in the analyses compared to those who answered ‘don't know’ is provided in the supporting information.

## 4 DISCUSSION

In this sample of 5835 people LWBC, only 31% perceived a need to improve their diet while 60% did not. In both models, individuals who were younger, female, not of white ethnicity, not married/cohabiting, and had received some dietary advice were more likely to perceive a need to improve their diet. Without accounting for dietary intake, participants with a higher BMI were more likely to perceive a need to improve their diet. On the other hand, where BMI was not included in the model, participants who reported having two or more comorbidities, met the WCRF recommendations for fruit and vegetables, fat and sugar were less likely to perceive a need to improve their diet.

This study supports previous research reporting an association between age, sex, ethnicity and marital status and diet‐related perceptions,^38^ but diverges from studies reporting associations between education and dietary perception.^39^ The association between older age and a reduced likelihood of perceiving a need to improve diet aligns with previous reports of higher self‐ratings of dietary quality in older age groups,^38^, ^39^ while the greater likelihood of females perceiving a need to improve diet in the dietary components model diverges from reported similarities across men and women in perceptions of dietary healthfulness.^40^, ^41^ This discrepancy may be attributed to the difference in asking participants to consider whether they need to improve their diet, which is distinct from rating the healthfulness of their diet.^38^, ^40^ In their study of people LWBC, Xue et al. asked participants to rate how healthy their diet was on a scale of one to five, while Batis et al. asked participants a closed binary question of whether they considered their diet to be healthy.^38^, ^40^ These assessments differ from the current study's question specifically asking about whether improvement to diet is needed and suggests that although men and women may assess the quality of their diets similarly, this does not translate equally across sexes into perceiving a need to change. While previous research has demonstrated sex differences in changes to diet in people diagnosed with colorectal cancer, with a higher prevalence of dietary changes alone reported by males and a higher prevalence of both dietary changes and supplement use reported by females,^54^ the present study's findings on perceiving a need to change are novel. Our results support previous findings in the general population that women place higher importance on diet and healthy eating than males,^55^ and extend these findings to people LWBC. In the dietary components model, participants experiencing more comorbidities were more likely to believe in a need to improve their diet. Heuchan et al. found a similar association between the number of comorbidities and perceiving a need to lose weight and suggested that other illness care pathways may help to raise awareness of weight status.^56^ This may also be the case for dietary information and accordingly, experiencing comorbid conditions may mitigate the effects of an unmet need for dietary advice that is reported in people LWBC.^30^, ^57^ Similarly, this study extends findings of an association between BMI and perceptions of dietary quality by demonstrating that people LWBC with overweight or obesity are more likely to perceive a need to improve their diet. However given that people with overweight and obesity are also at heightened risk for comorbidities, future research should seek to better understand the relationships between comorbidities, BMI, actual dietary intake and dietary change perceptions.^58^ Additionally, stigma may be an important mediating factor in the relationship between BMI and dietary change perceptions. Oversimplification of the causes of obesity means that the majority of people in the UK believe that individuals with obesity are themselves fully responsible for their dietary intake and heavier weight.^59^, ^60^ Internalisations of this belief could drive people with higher BMIs to perceive a need to improve their diet regardless of actual diet quality. Future research should aim to examine the potential mediating role of BMI on perceiving a need to change diet by using prospective designs to further investigate the causal pathway underlying the association between dietary intake and perceiving a need to change diet.

Previous research has identified perceiving one's diet to already be healthy enough as a formidable barrier in trying to encourage people to eat a healthier diet^61^, ^62^ and although people tend to be aware of nutritional guidelines, they appear to not perceive these to be relevant to their own diet.^61^ This could reflect misevaluations of their own diet or even a lack of belief in the role of diet in improving outcomes.^32^ Xue et al. reported low conformance between perceived and actual dietary quality in people LWBC, where 56% of participants overrated the healthfulness of their diet and overrating was associated with an increased intake of ‘empty calories’ from added sugars and fats.^38^ The current study shows more promising accuracy in dietary evaluations by demonstrating that participants correctly perceived a need to improve diet, where recommendations for sugar, fat and fruit and vegetables were not met. This may be attributed to large‐scale messaging about low‐fat and low‐calorie alternatives and the importance of 5‐a‐day targets.^63^

However, this finding also underscores the need to increase knowledge of the other dietary recommendations in people LWBC as it indicates that people rely on fruit and vegetable, sugar and fat consumption, when evaluating dietary quality, without consideration for other important dietary components Not knowing what constitutes a healthy diet is well documented in the literature, as well as reports of inadequate provision of dietary information after diagnosis within the cancer care pathway.^31^, ^64^ In this study, 58% of participants reported not receiving any dietary advice from their healthcare professionals. Receiving dietary advice was associated with perceiving a need to improve diet, suggesting that this may be an effective way to heighten awareness of dietary quality in this population. As a result of this unmet need for advice and information, many people LWBC report seeking information from other sources and the accessibility of unregulated online health information is concerning as this is not always evidence‐based and could be potentially harmful.^29^, ^30^, ^31^ Interestingly, adherence was highest for red meat and alcohol consumption, but these were not related to perceiving no need to change diet, suggesting that people follow these guidelines without equating them to having a healthy diet.^65^ Future dietary interventions should aim to promote the consideration of all dietary components together in choosing a healthy diet, particularly to reduce instances where people inaccurately believe that their diet is already healthy enough, based only on meeting the more widely known recommendations.

Strengths of this study include the large sample size of 5835 people LWBC, the use of multiple imputation to reduce the impact of bias related to missing information,^66^ and the novel exploration of the factors that influence perceiving a need to improve diet. However, these findings may not be directly generalisable to other cancer populations. Although findings were broadly similar among our subgroups, participants with colorectal cancer demonstrated some differences related to education level and a stronger association between ethnicity and perceiving a need to improve diet than in breast and prostate cancer participants. Future research should aim to provide a more comprehensive investigation of these differences. Other limitations include that this sample was not ethnically diverse, with 90% of participants identifying as white. Additionally, there were insufficient numbers in the ‘don't know’ group to be included in the regression analyses and there were some differences between this group and those who gave a ‘yes’ or ‘no’ answer to the dietary perception variable. Those who answered ‘yes’ or ‘no’ were younger and comprised a higher proportion of people of white ethnicity and people who were married/cohabiting. This group also demonstrated a higher level of education, fewer comorbidities, a higher proportion of people meeting the recommendations for fruit and vegetables, red meat, and sugar, and a lower proportion of people with an obese BMI. The results of this study should therefore be considered within this context and might therefore misrepresent participants who are, for example, less educated and this group may already be at higher risk of disease.^67^, ^68^ Furthermore, although similar findings were observed for both the imputed and non‐imputed data, completer participants tended to be younger, more educated, with less comorbidities, met more dietary recommendations and more reported having received dietary advice than non‐completers. Another limitation was that self‐report was used to assess diet and BMI, which may be prone to biases such as social desirability, where people respond to questions in a way that presents them in a more favourable light than what is objectively accurate.^65^, ^69^, ^70^ For instance, people tend to under‐report and over‐report certain foods based on perceived healthiness.^65^, ^70^ However, face‐to‐face methods of assessment are more costly and do not allow for the same sample size to be acquired.^71^ Lastly, the generalisability of these findings may be threatened by a selection bias, where previous research has indicated that people who agree to participate in questionnaires about their health behaviours tend to exhibit a stronger interest in their own health and improving their behaviours.^72^, ^73^, ^74^ In this study, 43% of people sent the initial letter responded and the results should be considered with the acknowledgement that this sample may not be representative of all people LWBC.

## 5 CONCLUSION

Despite a large proportion of people LWBC not meeting dietary recommendations, only 31% perceived a need to improve their diet. The results of this study can therefore be considered to align with previous reports of misperceptions in dietary quality.^33^, ^38^ Qualitative research may help provide an explanation for what is driving these perceptions, as Beeken et al.'s interviews with people LWBC revealed that if people LWBC had already made some changes to their diet, they may not perceive a need to continue to make changes, despite still not meeting the recommendations. Targeted interventions may be required to improve the accuracy of perceptions among certain groups, including older people and men as these groups were less likely to perceive a need to improve their diet. Education around the different dietary factors that contribute to a ‘healthy diet’, including red meat and alcohol intake, may also encourage perceptions that are more accurate. Improving the accuracy of perceptions about diet alongside behaviour change interventions could help individuals LWBC to improve their dietary intake and enhance their long‐term health.

## AUTHOR CONTRIBUTIONS

**Susan Smith:** Formal analysis (equal); methodology (equal); writing – original draft (equal); writing – review and editing (equal). **Abi Fisher:** Funding acquisition (equal); methodology (equal); supervision (equal); writing – review and editing (equal). **Phillippa J. Lally:** Data curation (equal); writing – review and editing (equal). **Helen A. Croker:** Writing – review and editing (equal). **Anna Roberts:** Data curation (equal); writing – review and editing (equal). **Rana E. Conway:** Methodology (equal); supervision (equal); writing – review and editing (equal). **Rebecca J. Beeken:** Conceptualization (equal); funding acquisition (equal); methodology (equal); supervision (equal); writing – review and editing (equal).

## FUNDING INFORMATION

Cancer Research UK C43975/A27498.

## CONFLICT OF INTEREST STATEMENT

The authors made no disclosures.

## PRECIS

Despite published dietary recommendations, we found that many people living with and beyond cancer do not follow a healthy diet and only 31% perceived a need to improve their diet. Being younger, female, not of white ethnicity, not married/cohabiting, having more comorbidities, having a higher body mass index, and not meeting various dietary recommendations were associated with perceiving a need to improve diet.

## Supporting information


Data S1.

## Data Availability

The data sets generated during and/or analysed during this study are available from the corresponding author on reasonable request.
