# Supplementary material for: Perceiving a need for dietary change in adults living with and beyond cancer: A cross‐sectional study
Source: Cancer Med. 2024 Mar 8;13(4):e7073. doi: 10.1002/cam4.7073 (PMC10922024; doi:10.1002/cam4.7073)
Supplement: Supplementary file 1 — Data S1. [file CAM4-13-e7073-s001.docx]

**Supporting information**

**Stratified analyses**

| **Supplementary table 1**  Pooled multivariate logistic regression analysis for the association between Body Mass Index (BMI) and perceiving a need to improve diet in breast cancer participants (n=2553)^†^ | | | |
| --- | --- | --- | --- |
|  | **OR** | **95CI** | ***p*** |
| **Age** | 0.95 | 0.94-0.96 | <.001* |
| **Education level** |  |  |  |
| None | 1 |  |  |
| GCSE/vocational | 1.16 | 0.90-1.49 | .247 |
| A-level | 1.03 | 0.74-1.42 | .878 |
| Degree and above | 1.16 | 0.89-1.52 | .281 |
| **Ethnicity** |  |  |  |
| White | 1 |  |  |
| Any other ethnicity | 1.67 | 1.25-2.21 | <.001* |
| **Marital status** |  |  |  |
| Married/cohabiting | 1 |  |  |
| Separated/divorced/widowed/single | 1.28 | 1.06-1.54 | .009* |
| **Cancer spread** |  |  |  |
| Yes | 1 |  |  |
| No | 1.07 | 0.81-1.43 | .630 |
| **Months since cancer diagnosis** | 1.00^‡^ | 0.99-1.00 | .300 |
| **Number of comorbid conditions** |  |  |  |
| None | 1 |  |  |
| 1 | 1.07 | 0.86-1.32 | .554 |
| 2 | 1.10 | 0.85-1.43 | .461 |
| 3 or more | 1.53 | 1.14-2.03 | .005* |
| **Receipt of dietary advice** |  |  |  |
| No advice received | 1 |  |  |
| Advice received | 1.33 | 1.10-1.61 | .003* |
| **BMI** |  |  |  |
| Healthy/underweight | 1 |  |  |
| Overweight | 1.68 | 1.37-2.07 | <.001* |
| Obese | 2.70 | 2.12-3.42 | <.001* |
| ^†^ ‘Don’t know’ cases excluded from analysis. Males excluded from the analysis (n=25). ^‡^0.996 rounded up. *indicates statistical significance at *p* <.05. Abbreviations: OR = odds ratio; 95CI = 95% confidence interval; GCSE = General Certificate of Secondary Education; A-level = General Certificate of Education Advanced Level; BMI = body mass index. | | | |

| **Supplementary table 2**  Pooled multivariate logistic regression analysis for the association between dietary components and perceiving a need to improve diet in breast cancer participants (n=2553)^†^ | | | |
| --- | --- | --- | --- |
|  | **OR** | **95CI** | ***p*** |
| **Age** | 0.95 | 0.94-0.96 | <.001* |
| **Education level** |  |  |  |
| None | 1 |  |  |
| GCSE/vocational | 1.17 | 0.91-1.51 | .222 |
| A level | 1.07 | 0.77-1.49 | .682 |
| Degree and above | 1.26 | 0.96-1.65 | .100 |
| **Ethnicity** |  |  |  |
| White | 1 |  |  |
| Any other ethnicity | 1.78 | 1.32-2.39 | <.001* |
| **Marital status** |  |  |  |
| Married/cohabiting | 1 |  |  |
| Separated/divorced/widowed/single | 1.24 | 1.03-1.50 | .024 |
| **Cancer spread** |  |  |  |
| Yes | 1 |  |  |
| No | 1.10 | 0.81-1.47 | .549 |
| **Months since diagnosis** | 0.99 | 0.99-1.00 | .065 |
| **Number of comorbid conditions** |  |  |  |
| None | 1 |  |  |
| 1 | 1.11 | 0.90-1.38 | .335 |
| 2 | 1.20 | 0.93-1.56 | .169 |
| 3 or more | 1.74 | 1.30-2.33 | <.001 |
| **Dietary advice received** |  |  |  |
| No advice received | 1 |  |  |
| Advice received | 1.38 | 1.14-1.67 | <.001* |
| **WCRF/AICR fibre** |  |  |  |
| Not meeting | 1 |  |  |
| Meeting | 1.06 | 0.81-1.39 | .664 |
| **WCRF/AICR fruit and vegetables** |  |  |  |
| Not meeting | 1 |  |  |
| Meeting | 0.44 | 0.36-0.53 | <.001* |
| **WCRF/AICR red meat** |  |  |  |
| Not meeting | 1 |  |  |
| Meeting | 1.34 | 0.79-2.25 | .255 |
| **WCRF/AICR processed meat** |  |  |  |
| Not meeting | 1 |  |  |
| Meeting | 0.98 | 0.81-1.19 | .856 |
| **WCRF/AICR fat** |  |  |  |
| Not meeting | 1 |  |  |
| Meeting | 0.59 | 0.47-0.73 | <.001* |
| **WCRF/AICR sugar** |  |  |  |
| Not meeting | 1 |  |  |
| Meeting | 0.84 | 0.70-1.01 | .062 |
| **WCRF/AICR alcohol** |  |  |  |
| Not meeting | 1 |  |  |
| Meeting | 0.98 | 0.68-1.41 | .902 |
| ^†^‘Don’t know’ cases excluded from analysis. Males were excluded from the analysis (n=25). *indicates statistical significance at *p* <.05. Abbreviations: OR = odds ratio; 95CI = 95% confidence interval; GCSE = General Certificate of Secondary Education; ^§^A-level = General Secondary School Advanced Level; WCRF/AICR = World Cancer Research Fund and American Institute for Cancer Research recommendations. | | | |

| **Supplementary table 3**  Pooled multivariate logistic regression analysis for the association between Body Mass Index (BMI) and perceiving a need to improve diet in prostate cancer participants (n=1692)^†^ | | | |
| --- | --- | --- | --- |
|  | **OR** | **95CI** | ***p*** |
| **Age** | 0.93 | 0.92-0.95 | <.001* |
| **Education level** |  |  |  |
| None | 1 |  |  |
| GCSE/vocational | 1.06 | 0.74-1.51 | .754 |
| A-level | 1.15 | 0.73-1.83 | .540 |
| Degree and above | 0.94 | 0.66-1.35 | .739 |
| **Ethnicity** |  |  |  |
| White | 1 |  |  |
| Any other ethnicity | 1.46 | 1.02-2.09 | .039* |
| **Marital status** |  |  |  |
| Married/cohabiting | 1 |  |  |
| Separated/divorced/widowed/single | 1.44 | 1.10-1.89 | .008* |
| **Cancer spread** |  |  |  |
| Yes | 1 |  |  |
| No | 1.04 | 0.65-1.67 | .854 |
| **Months since cancer diagnosis** | 1.00^‡^ | 0.99-1.00 | .375 |
| **Number of comorbid conditions** |  |  |  |
| None | 1 |  |  |
| 1 | 0.93 | 0.70-1.23 | .598 |
| 2 | 1.25 | 0.90-1.73 | .188 |
| 3 or more | 0.99 | 0.68-1.46 | .972 |
| **Receipt of dietary advice** |  |  |  |
| No advice received | 1 |  |  |
| Advice received | 1.93 | 1.53-2.45 | <.001* |
| **BMI** |  |  |  |
| Healthy/underweight | 1 |  |  |
| Overweight | 1.44 | 1.09-1.90 | .011* |
| Obese | 2.81 | 2.03-3.89 | <.001* |
| ^†^ ‘Don’t know’ cases excluded from analysis. ^‡^0.997 rounded up. *indicates statistical significance at *p* <.05 Abbreviations: OR = odds ratio; 95CI = 95% confidence interval; GCSE = General Certificate of Secondary Education; A-level = General Certificate of Education Advanced Level; BMI = body mass index. | | | |

| **Supplementary table 4**  Pooled multivariate logistic regression analysis for the association between dietary components and perceiving a need to improve diet in prostate cancer participants (n=1692)^†^ | | | |
| --- | --- | --- | --- |
|  | **OR** | **95CI** | ***p*** |
| **Age** | 0.92 | 0.91-0.94 | <.001* |
| **Education level** |  |  |  |
| None | 1 |  |  |
| GCSE/vocational | 1.09 | 0.76-1.57 | .637 |
| A level | 1.15 | 0.72-1.83 | .560 |
| Degree and above | 0.96 | 0.66-1.39 | .836 |
| **Ethnicity** |  |  |  |
| White | 1 |  |  |
| Any other ethnicity | 1.47 | 1.01-2.15 | .045* |
| **Marital status** |  |  |  |
| Married/cohabiting | 1 |  |  |
| Separated/divorced/widowed/single | 1.38 | 1.05-1.80 | .021* |
| **Cancer spread** |  |  |  |
| Yes | 1 |  |  |
| No | 1.08 | 0.67-1.73 | .751 |
| **Months since diagnosis** | 1.00^‡^ | 0.99-1.01 | .454 |
| **Number of comorbid conditions** |  |  |  |
| None | 1 |  |  |
| 1 | 0.98 | 0.74-1.30 | .875 |
| 2 | 1.36 | 0.98-1.89 | .068 |
| 3 or more | 1.19 | 0.82-1.73 | .364 |
| **Dietary advice received** |  |  |  |
| No advice received | 1 |  |  |
| Advice received | 2.01 | 1.59-2.54 | <.001* |
| **WCRF/AICR fibre** |  |  |  |
| Not meeting | 1 |  |  |
| Meeting | 0.82 | 0.59-1.14 | .243 |
| **WCRF/AICR fruit and vegetables** |  |  |  |
| Not meeting | 1 |  |  |
| Meeting | 0.61 | 0.45-0.83 | .002* |
| **WCRF/AICR red meat** |  |  |  |
| Not meeting | 1 |  |  |
| Meeting | 1.37 | 0.84-2.24 | .208 |
| **WCRF/AICR processed meat** |  |  |  |
| Not meeting | 1 |  |  |
| Meeting | 0.91 | 0.70-1.17 | .458 |
| **WCRF/AICR fat** |  |  |  |
| Not meeting | 1 |  |  |
| Meeting | 0.68 | 0.50-0.92 | .014* |
| **WCRF/AICR sugar** |  |  |  |
| Not meeting | 1 |  |  |
| Meeting | 0.92 | 0.72-1.16 | .472 |
| **WCRF/AICR alcohol** |  |  |  |
| Not meeting | 1 |  |  |
| Meeting | 1.19 | 0.88-1.61 | .258 |
| ^†^‘Don’t know’ cases excluded from analysis. ^‡^0.997 rounded up. *indicates statistical significance at *p* <.05. Abbreviations: OR = odds ratio; 95CI = 95% confidence interval; GCSE = General Certificate of Secondary Education; A-level = General Secondary School Advanced Level; WCRF/AICR = World Cancer Research Fund and American Institute for Cancer Research recommendations. | | | |

| **Supplementary table 5**  Pooled multivariate logistic regression analysis for the association between Body Mass Index (BMI) and perceiving a need to improve diet in colorectal cancer participants (n=1089)^†^ | | | |
| --- | --- | --- | --- |
|  | **OR** | **95CI** | ***p*** |
| **Age** | 0.96 | 0.94-0.97 | <.001* |
| **Sex** |  |  |  |
| Male | 1 |  |  |
| Female | 1.62 | 1.19-2.20 | .002* |
| **Education level** |  |  |  |
| None | 1 |  |  |
| GCSE/vocational | 0.66 | 0.45-0.99 | .042 |
| A-level | 0.90 | 0.53-1.51 | .677 |
| Degree and above | 0.73 | 0.48-1.09 | .123 |
| **Ethnicity** |  |  |  |
| White | 1 |  |  |
| Any other ethnicity | 3.34 | 1.80-6.22 | <.001* |
| **Marital status** |  |  |  |
| Married/cohabiting | 1 |  |  |
| Separated/divorced/widowed/single | 1.29 | 0.93-1.80 | .130 |
| **Cancer spread** |  |  |  |
| Yes | 1 |  |  |
| No | 1.24 | 0.80-1.91 | .332 |
| **Months since cancer diagnosis** | 1.01 | 1.00-1.02 | .127 |
| **Number of comorbid conditions** |  |  |  |
| None | 1 |  |  |
| 1 | 0.80 | 0.55-1.15 | .225 |
| 2 | 1.29 | 0.83-2.02 | .260 |
| 3 or more | 0.91 | 0.58-1.44 | .693 |
| **Receipt of dietary advice** |  |  |  |
| No advice received | 1 |  |  |
| Advice received | 1.78 | 1.32-2.41 | <.001* |
| **BMI** |  |  |  |
| Healthy/underweight | 1 |  |  |
| Overweight | 1.39 | 0.96-2.02 | .077 |
| Obese | 2.70 | 1.81-4.01 | <.001* |
| ^†^ ‘Don’t know’ cases excluded from analysis. *indicates statistical significance at *p* <.05. Abbreviations: OR = odds ratio; 95CI = 95% confidence interval; GCSE = General Certificate of Secondary Education; A-level = General Certificate of Education Advanced Level; BMI = body mass index. | | | |

| **Supplementary table 6**  Pooled multivariate logistic regression analysis for the association between dietary components and perceiving a need to improve diet in colorectal cancer participants (n=1089)^†^ | | | |
| --- | --- | --- | --- |
|  | **OR** | **95CI** | ***p*** |
| **Age** | 0.95 | 0.94-0.97 | <.001* |
| **Sex** |  |  |  |
| Male | 1 |  |  |
| Female | 1.70 | 1.23-2.34 | .001* |
| **Education level** |  |  |  |
| None | 1 |  |  |
| GCSE/vocational | 0.66 | 0.44-0.99 | .043* |
| A level | 1.00^¶^ | 0.59-1.69 | .987 |
| Degree and above | 0.76 | 0.50-1.15 | .193 |
| **Ethnicity** |  |  |  |
| White | 1 |  |  |
| Any other ethnicity | 3.32 | 1.76-6.28 | <.001* |
| **Marital status** |  |  |  |
| Married/cohabiting | 1 |  |  |
| Separated/divorced/widowed/single | 1.28 | 0.92-1.78 | .141 |
| **Cancer spread** |  |  |  |
| Yes | 1 |  |  |
| No | 1.27 | 0.82-1.98 | .283 |
| **Months since diagnosis** | 1.01 | 1.00-1.02 | .203 |
| **Number of comorbid conditions** |  |  |  |
| None | 1 |  |  |
| 1 | 0.91 | 0.63-1.33 | .638 |
| 2 | 1.64 | 1.05-2.57 | .030* |
| 3 or more | 1.15 | 0.73-1.81 | .540 |
| **Dietary advice received** |  |  |  |
| No advice received | 1 |  |  |
| Advice received | 1.95 | 1.44-2.63 | <.001* |
| **WCRF/AICR fibre** |  |  |  |
| Not meeting | 1 |  |  |
| Meeting | 0.77 | 0.49-1.20 | .243 |
| **WCRF/AICR fruit and vegetables** |  |  |  |
| Not meeting | 1 |  |  |
| Meeting | 0.41 | 0.28-0.60 | <.001* |
| **WCRF/AICR red meat** |  |  |  |
| Not meeting | 1 |  |  |
| Meeting | 1.26 | 0.64-2.50 | .492 |
| **WCRF/AICR processed meat** |  |  |  |
| Not meeting | 1 |  |  |
| Meeting | 0.83 | 0.59-1.15 | .254 |
| **WCRF/AICR fat** |  |  |  |
| Not meeting | 1 |  |  |
| Meeting | 0.88 | 0.63-1.22 | .434 |
| **WCRF/AICR sugar** |  |  |  |
| Not meeting | 1 |  |  |
| Meeting | 0.81 | 0.60-1.09 | .163 |
| **WCRF/AICR alcohol** |  |  |  |
| Not meeting | 1 |  |  |
| Meeting | 1.34 | 0.83-2.14 | .229 |
| ^†^‘Don’t know’ cases excluded from analysis. *indicates statistical significance at *p* <.05. Abbreviations: OR = odds ratio; 95CI = 95% confidence interval; GCSE = General Certificate of Secondary Education; A-level = General Secondary School Advanced Level; WCRF/AICR = World Cancer Research Fund and American Institute for Cancer Research recommendations. | | | |

**Comparison of completers and non-completers of key variables**

*Completers consisted of a higher proportion of females and breast cancer patients than non-completers. Completers tended to be younger, had a higher level of education and fewer comorbidities than non-completers. A higher proportion of completers indicated that they had received dietary advice than in the non-completers. Completers demonstrated higher adherence to the World Cancer Research Fund recommendations.*

| **Supplementary table 7**  Descriptive statistics of key variables for completer and non-completer participants | | | |
| --- | --- | --- | --- |
|  | **Completers**  **(*n*=2005)** | **Non-completers** **(*n*=3830)** | **All (*n*=5835)** |
| **Perception of need for dietary change (%)** |  |  |  |
| Need to improve | 33.2 | 29.5 | 30.7 |
| No need to change | 61.0 | 59.7 | 60.2 |
| Don’t know | 5.8 | 9.2 | 8.0 |
| **Age, *Mean (SD)*** | 64.0 (11.8) | 69.2 (11.4) | 67.4 (11.8) |
| **Sex (%)** |  |  |  |
| Male | 40.7 | 45.4 | 43.8 |
| Female | 59.3 | 54.2 | 56.0 |
| **Ethnicity (%)** |  |  |  |
| White | 93.7 | 88.0 | 90.0 |
| Any other ethnicity | 6.3 | 11.2 | 9.5 |
| **Highest Level of Education (%)** |  |  |  |
| None | 21.8 | 33.2 | 24.0 |
| GCSE/vocational | 34.2 | 24.2 | 27.6 |
| A level | 12.6 | 8.7 | 10.0 |
| Degree | 31.4 | 19.6 | 23.6 |
| **Marital Status (%)** |  |  |  |
| Married/cohabiting | 74.5 | 66.4 | 69.2 |
| Separated/divorces/widowed/single | 25.5 | 33.2 | 30.5 |
| **BMI (%)** |  |  |  |
| Underweight/healthy | 38.7 | 33.1 | 35.0 |
| Overweight | 40.0 | 37.8 | 38.5 |
| Obese | 21.4 | 20.4 | 20.7 |
| **Cancer Type (%)** |  |  |  |
| Breast | 52.3 | 45.4 | 47.7 |
| Prostate | 28.2 | 33.3 | 31.5 |
| Colorectal | 19.6 | 21.4 | 20.7 |
| **Time since diagnosis, *Mean (SD)*** | 35.4 (13.2) | 36.5 (14.2) | 36.1 (13.8) |
| **Treatment type (ticked yes)** |  |  |  |
| Surgery | 74.6 | 67.1 | 69.7 |
| Radiotherapy | 59.5 | 56.3 | 57.4 |
| Chemotherapy | 35.6 | 29.0 | 31.3 |
| Hormone therapy | 36.8 | 30.2 | 32.5 |
| Active surveillance | 17.3 | 17.5 | 17.4 |
| Biological therapies | 2.5 | 1.9 | 2.1 |
| Other treatment | 0.9 | 0.7 | 0.8 |
| No treatment | 0.9 | 2.5 | 1.9 |
| **Cancer Spread (%)** |  |  |  |
| Yes | 11.9 | 8.3 | 9.6 |
| No | 88.1 | 71.3 | 77.1 |
| **Number of Comorbidities (%)** |  |  |  |
| 0 | 36.0 | 29.4 | 31.7 |
| 1 | 33.4 | 34.5 | 34.1 |
| 2 | 18.3 | 19.7 | 19.2 |
| 3 or more | 12.3 | 16.4 | 15 |
| **Receipt of dietary advice** |  |  |  |
| No advice received | 70.0 | 50.9 | 57.4 |
| Advice received | 30.0 | 23.1 | 35.1 |
| **WCRF/AICR fibre (%)** |  |  |  |
| Not meeting | 86.6 | 58.8 | 85.4 |
| Meeting | 13.4 | 10.4 | 14.6 |
| **WCRF/AICR fruit and vegetables (%)** |  |  |  |
| Not meeting | 67.5 | 69.3 | 67.1 |
| Meeting | 32.5 | 26.3 | 11.4 |
| **WCRF/AICR red meat (%)** |  |  |  |
| Not meeting | 2.1 | 2.5 | 2.4 |
| Meeting | 97.9 | 80.2 | 86.3 |
| **WCRF/AICR processed meat (%)** |  |  |  |
| Not meeting | 51.2 | 47.9 | 49.0 |
| Meeting | 48.8 | 43.4 | 45.2 |
| **WCRF/AICR sugar (%)** |  |  |  |
| Not meeting | 45.4 | 45.7 | 45.6 |
| Meeting | 54.6 | 41.8 | 46.2 |
| **WCRF/AICR fat (%)** |  |  |  |
| Not meeting | 42.4 | 24.0 | 30.3 |
| Meeting | 57.6 | 29.9 | 39.4 |
| **WCRF/AICR alcohol (%)** |  |  |  |
| Not meeting | 14.0 | 11.3 | 12.2 |
| Meeting | 86.0 | 81.6 | 83.1 |
| Abbreviations: SD = standard deviation; GCSE = General Certificate of Secondary Education; A-level = General Secondary School Advanced Level; BMI = body mass index; WCRF/AICR = World Cancer Research Fund and American Institute for Cancer Research recommendations. | | | |

**Logistic regression analyses in the original data**

| **Supplementary table 8**  Multivariate logistic regression analysis for the association between Body Mass Index (BMI) and perceiving a need to improve diet in the original data (n=3356)^†^ | | | |
| --- | --- | --- | --- |
|  | **OR** | **95CI** | ***p*** |
| **Age** | 0.96 | 0.95-0.96 | <.001* |
| **Sex** |  |  |  |
| Male | 1 |  |  |
| Female | 1.27 | 1.07-1.52 | .008 |
| **Education level** |  |  |  |
| None | 1 |  |  |
| GCSE/vocational | 1.13 | 0.91-1.39 | .272 |
| A-level | 1.04 | 0.78-1.38 | .795 |
| Degree and above | 1.12 | 0.90-1.41 | .307 |
| **Ethnicity** |  |  |  |
| White | 1 |  |  |
| Any other ethnicity | 1.56 | 1.19-2.05 | .001* |
| **Marital status** |  |  |  |
| Married/cohabiting | 1 |  |  |
| Separated/divorced/widowed/single | 1.25 | 1.06-1.49 | .012* |
| **Cancer spread** |  |  |  |
| Yes | 1 |  |  |
| No | 1.16 | 0.91-1.48 | .238 |
| **Months since cancer diagnosis** | 1.00^‡^ | 1.00-1.01 | .222 |
| **Number of comorbid conditions** |  |  |  |
| None | 1 |  |  |
| 1 | 0.86 | 0.74-1.07 | .199 |
| 2 | 1.21 | 0.97-1.52 | .095 |
| 3 or more | 1.17 | 0.90-1.52 | .246 |
| **Receipt of dietary advice** |  |  |  |
| No advice received | 1 |  |  |
| Advice received | 1.36 | 1.15-1.61 | <.001* |
| **BMI** |  |  |  |
| Healthy/underweight | 1 |  |  |
| Overweight | 1.52 | 1.27-1.81 | <.001* |
| Obese | 2.89 | 2.35-3.56 | <.001* |
| ^†^ ‘Don’t know’ cases excluded from analysis. ^‡^1.003 rounded down. *indicates statistical significance at *p* <.05. Abbreviations: OR = odds ratio; 95CI = 95% confidence interval; GCSE = General Certificate of Secondary Education; A-level = General Certificate of Education Advanced Level; BMI = body mass index. | | | |

| **Supplementary table 9**  Multivariate logistic regression analysis for the association between dietary components and perceiving a need to improve diet in the original data (n=2249)^†^ | | | |
| --- | --- | --- | --- |
|  | **OR** | **95CI** | ***p*** |
| **Age** | 0.95 | 0.94-0.96 | <.001* |
| **Sex** |  |  |  |
| Male | 1 |  |  |
| Female | 1.24 | 0.99-1.56 | .057 |
| **Education level** |  |  |  |
| None | 1 |  |  |
| GCSE/vocational | 1.10 | 0.84-1.45 | .486 |
| A level | 1.11 | 0.78-1.56 | .564 |
| Degree and above | 1.24 | 0.93-1.65 | .138 |
| **Ethnicity** |  |  |  |
| White | 1 |  |  |
| Any other ethnicity | 2.10 | 1.47-3.10 | <.001* |
| **Marital status** |  |  |  |
| Married/cohabiting | 1 |  |  |
| Separated/divorced/widowed/single | 1.36 | 1.09-1.69 | .006 |
| **Cancer spread** |  |  |  |
| Yes | 1 |  |  |
| No | 1.22 | 0.91-1.64 | .184 |
| **Months since diagnosis** | 1.00^‡^ | 1.00-1.01 | .553 |
| **Number of comorbid conditions** |  |  |  |
| None | 1 |  |  |
| 1 | 0.82 | 0.65-1.02 | .079 |
| 2 | 1.23 | 0.93-1.62 | .152 |
| 3 or more | 1.52 | 1.11-2.08 | .010* |
| **Dietary advice received** |  |  |  |
| No advice received | 1 |  |  |
| Advice received | 1.39 | 1.13-1.70 | .002* |
| **WCRF/AICR fibre** |  |  |  |
| Not meeting | 1 |  |  |
| Meeting | 0.82 | 0.61-1.10 | <.001* |
| **WCRF/AICR fruit and vegetables** |  |  |  |
| Not meeting | 1 |  |  |
| Meeting | 0.44 | 0.36-0.55 | <.001* |
| **WCRF/AICR red meat** |  |  |  |
| Not meeting | 1 |  |  |
| Meeting | 1.83 | 0.92-3.63 | .088 |
| **WCRF/AICR processed meat** |  |  |  |
| Not meeting | 1 |  |  |
| Meeting | 0.88 | 0.72-1.08 | .218 |
| **WCRF/AICR fat** |  |  |  |
| Not meeting | 1 |  |  |
| Meeting | 0.64 | 0.52-0.79 | <.001* |
| **WCRF/AICR sugar** |  |  |  |
| Not meeting | 1 |  |  |
| Meeting | 0.76 | 0.63-0.93 | .006* |
| **WCRF/AICR alcohol** |  |  |  |
| Not meeting | 1 |  |  |
| Meeting | 1.26 | 0.96-1.66 | .102 |
| ^†^‘Don’t know’ cases excluded from analysis. ^‡^0.997 rounded up. *indicates statistical significance at *p* <.05. Abbreviations: OR = odds ratio; 95CI = 95% confidence interval; GCSE = General Certificate of Secondary Education; A-level = General Certificate of Education Advanced Level; WCRF/AICR = World Cancer Research Fund and American Institute for Cancer Research recommendations. | | | |

| **Supplementary table 10.**  Chi squared analyses comparing people answered ‘yes’ or no’ and those who answered ‘don’t know’ in the original data | | | |
| --- | --- | --- | --- |
|  | **‘Yes’ or ‘No’ (n=5304)** | **‘Don’t know’ (n=468)** | ***p*** |
| **Age, *Mean (SD)*** | 67.24 (11.7) | 69.07 (12.3) | .001 |
| **Sex (%)** |  |  |  |
| Male | 43.8 | 44.8 |  |
| Female | 56.2 | 55.2 | .682 |
| **Ethnicity n(%)** |  |  |  |
| White | 90.7 | 87.5 |  |
| Any other ethnicity | 9.3 | 12.5 | .026* |
| **Highest Level of Education (%)** |  |  |  |
| None | 31.1 | 45.0 |  |
| GCSE/vocational | 30.9 | 26.0 |  |
| A-level | 11.1 | 10.5 |  |
| Degree | 26.9 | 18.6 | <.001* |
| **Marital Status (%)** |  |  |  |
| Married/cohabiting | 70.4 | 59.7 |  |
| Separated/divorces/widowed/single | 29.6 | 40.3 | <.001* |
| **BMI (%)** |  |  |  |
| Underweight/healthy | 37.4 | 32.9 |  |
| Overweight | 41.2 | 37.0 |  |
| Obese | 21.3 | 30.2 | <.001* |
| **Cancer Type (%)** |  |  |  |
| Breast | 48.1 | 44.4 |  |
| Prostate | 31.6 | 30.6 |  |
| Colorectal | 20.3 | 25.0 | .053 |
| **Time since diagnosis, *Mean (SD)*** | 13.9 | 13.4 | .112 |
| **Cancer Spread (%)** |  |  |  |
| Yes | 10.9 | 13.5 |  |
| No | 89.1 | 86.5 | .108 |
| **Number of Comorbidities (%)** |  |  |  |
| 0 | 32.2 | 25.0 |  |
| 1 | 34.5 | 30.6 |  |
| 2 | 18.7 | 23.9 |  |
| 3 or more | 14.5 | 20.5 | <.001* |
| **Receipt of dietary advice** |  |  |  |
| No advice received | 69.3 | 68.5 |  |
| Advice received | 30.7 | 113 | .758 |
| **WCRF/AICR fibre (%)** |  |  |  |
| Not meeting | 85.3 | 87.3 |  |
| Meeting | 14.7 | 12.7 | .299 |
| **WCRF/AICR fruit and vegetables (%)** |  |  |  |
| Not meeting | 69.6 | 83.6 |  |
| Meeting | 30.4 | 16.4 | <.001* |
| **WCRF/AICR red meat (%)** |  |  |  |
| Not meeting | 2.4 | 5.6 |  |
| Meeting | 97.6 | 94.4 | <.001* |
| **WCRF/AICR processed meat (%)** |  |  |  |
| Not meeting | 51.8 | 54.6 |  |
| Meeting | 48.2 | 45.4 | .266 |
| **WCRF/AICR sugar (%)** |  |  |  |
| Not meeting | 49.2 | 54.8 |  |
| Meeting | 50.8 | 45.2 | .031* |
| **WCRF/AICR fat (%)** |  |  |  |
| Not meeting | 43.4 | 45.3 |  |
| Meeting | 56.6 | 54.7 | .538 |
| **WCRF/AICR alcohol (%)** |  |  |  |
| Not meeting | 13.1 | 10.5 |  |
| Meeting | 86.9 | 89.5 | .125 |
| Abbreviations: SD = standard deviation; GCSE = General Certificate of Secondary Education; A-level = General Certificate of Education Advanced Level BMI = body mass index. WCRF/AICR = World Cancer Research Fund and American Institute for Cancer Research recommendations. | | | |
